# Supplementary material for: Modeling glioblastoma heterogeneity as a dynamic network of cell states
Source: Mol Syst Biol. 2021 Sep 16;17(9):e10105. doi: 10.15252/msb.202010105 (PMC8444284; doi:10.15252/msb.202010105)
Supplement: Supplementary file 6 — Source Data for Figure 5 [file MSB-17-e10105-s004.zip › Figure5A_sourcedata/GSEA_3071/hallmarks_stateII.GseaPreranked.1623742536692/gsea_report_for_na_neg_1623742536692.html]

Report for na\_neg 1623742536692 [GSEA]

| GS  follow link to MSigDB | GS DETAILS | SIZE | ES | NES | NOM p-val | FDR q-val | FWER p-val | RANK AT MAX | LEADING EDGE || 1 | HALLMARK\_KRAS\_SIGNALING\_UP | Details ... | 59 | -0.38 | -1.90 | 0.002 | 0.061 | 0.072 | 436 | tags=29%, list=11%, signal=32% |
| 2 | HALLMARK\_HEDGEHOG\_SIGNALING | Details ... | 19 | -0.48 | -1.73 | 0.035 | 0.113 | 0.243 | 970 | tags=58%, list=24%, signal=75% |
| 3 | HALLMARK\_MYOGENESIS | Details ... | 61 | -0.33 | -1.68 | 0.007 | 0.116 | 0.356 | 332 | tags=21%, list=8%, signal=23% |
| 4 | HALLMARK\_HEME\_METABOLISM | Details ... | 56 | -0.34 | -1.65 | 0.012 | 0.111 | 0.425 | 1143 | tags=50%, list=28%, signal=68% |
| 5 | HALLMARK\_TGF\_BETA\_SIGNALING | Details ... | 29 | -0.38 | -1.56 | 0.050 | 0.152 | 0.623 | 1071 | tags=55%, list=26%, signal=74% |
| 6 | HALLMARK\_KRAS\_SIGNALING\_DN | Details ... | 29 | -0.37 | -1.51 | 0.056 | 0.169 | 0.740 | 997 | tags=48%, list=24%, signal=63% |
| 7 | HALLMARK\_HYPOXIA | Details ... | 81 | -0.24 | -1.29 | 0.134 | 0.451 | 0.977 | 506 | tags=22%, list=12%, signal=25% |
| 8 | HALLMARK\_APOPTOSIS | Details ... | 57 | -0.26 | -1.28 | 0.168 | 0.417 | 0.980 | 539 | tags=26%, list=13%, signal=30% |
| 9 | HALLMARK\_OXIDATIVE\_PHOSPHORYLATION | Details ... | 69 | -0.23 | -1.19 | 0.221 | 0.546 | 0.999 | 1560 | tags=54%, list=38%, signal=85% |
| 10 | HALLMARK\_P53\_PATHWAY | Details ... | 70 | -0.23 | -1.14 | 0.276 | 0.600 | 1.000 | 519 | tags=21%, list=13%, signal=24% |
| 11 | HALLMARK\_XENOBIOTIC\_METABOLISM | Details ... | 47 | -0.23 | -1.05 | 0.399 | 0.744 | 1.000 | 436 | tags=21%, list=11%, signal=24% |
| 12 | HALLMARK\_COMPLEMENT | Details ... | 56 | -0.21 | -1.03 | 0.425 | 0.742 | 1.000 | 557 | tags=21%, list=14%, signal=24% |
| 13 | HALLMARK\_IL6\_JAK\_STAT3\_SIGNALING | Details ... | 15 | -0.29 | -1.00 | 0.485 | 0.756 | 1.000 | 954 | tags=47%, list=23%, signal=61% |
| 14 | HALLMARK\_CHOLESTEROL\_HOMEOSTASIS | Details ... | 29 | -0.24 | -0.99 | 0.477 | 0.707 | 1.000 | 587 | tags=21%, list=14%, signal=24% |
| 15 | HALLMARK\_COAGULATION | Details ... | 34 | -0.23 | -0.97 | 0.507 | 0.696 | 1.000 | 359 | tags=18%, list=9%, signal=19% |
| 16 | HALLMARK\_INTERFERON\_ALPHA\_RESPONSE | Details ... | 24 | -0.24 | -0.94 | 0.536 | 0.717 | 1.000 | 2471 | tags=88%, list=60%, signal=219% |
| 17 | HALLMARK\_PROTEIN\_SECRETION | Details ... | 32 | -0.21 | -0.87 | 0.653 | 0.813 | 1.000 | 1776 | tags=66%, list=43%, signal=115% |
| 18 | HALLMARK\_ADIPOGENESIS | Details ... | 56 | -0.16 | -0.77 | 0.789 | 0.922 | 1.000 | 631 | tags=18%, list=15%, signal=21% |
| 19 | HALLMARK\_FATTY\_ACID\_METABOLISM | Details ... | 43 | -0.16 | -0.72 | 0.832 | 0.942 | 1.000 | 555 | tags=14%, list=14%, signal=16% |
| 20 | HALLMARK\_REACTIVE\_OXYGEN\_SPECIES\_PATHWAY | Details ... | 20 | -0.18 | -0.67 | 0.869 | 0.940 | 1.000 | 422 | tags=15%, list=10%, signal=17% |
| 21 | HALLMARK\_BILE\_ACID\_METABOLISM |  | 22 | -0.17 | -0.64 | 0.893 | 0.924 | 1.000 | 534 | tags=18%, list=13%, signal=21% |
Table: Gene sets enriched in phenotype **na **[plain text format]****
